# Comparison of clinical outcome of decompression of suprascapular nerve at spinoglenoid notch for patients with posterosuperior massive rotator cuff tears and suprascapular neuropathy

**DOI:** 10.1186/s12891-021-04075-1

**Published:** 2021-02-18

**Authors:** Pu Yang, Chen Wang, Dongfang Zhang, Yi Zhang, Tengbo Yu, Chao Qi

**Affiliations:** Orthopaedic Center, the Affiliated Hospital of Qingdao University, NO.16 Jiangsu road, Qingdao, 266100 China

**Keywords:** Massive rotator cuff tear, Suprascapular nerve, Decompression, Spinoglenoid notch, Arthroscopy, Infraspinatus

## Abstract

**Purpose:**

In the present study, we aimed to determine whether decompression of suprascapular nerve (SSN) at the spinoglenoid notch could lead to a better functional outcome for the patients who underwent repairment of rotator cuff due to posterosupeior massive rotator cuff tear (MRCT) and suprascapular neuropathy.

**Methods:**

A total of 20 patients with posterosuperior MRCT and suprascapular neuropathy were analyzed in the present work. The preoperative magnetic resonance imaging (MRI) showed rotator cuff tear in supraspinatus and infraspinatus. All patients underwent arthroscopic rotator cuff repair. Patients were divided into two groups (group A: non-releasing, group B: releasing) according to whether the SSN at the spinoglenoid notch was decompressed. The modified University of California at Los Angeles shoulder rating scale (UCLA) and visual analog scale (VAS) questionnaire were adopted to assess the function of the affected shoulder preoperatively and 12 months after the operation. Electromyography (EMG) and nerve conduction study (NCS) were used to evaluate the nerve condition. Patients underwent MRI and EMG/NCS at 6 months after operation and last follow-up.

**Results:**

All patients were satisfied with the treatment. MRI showed that it was well-healed in 19 patients at 6 months after the operation. However, the fatty infiltration of supraspinatus and infraspinatus was not reversed. Only one patient in the non-releasing group showed the retear. The retear rate of group A and group B were 30% (3/10) and 20% (2/10) respectively at 12 months after the operation. One patient undergoing SSN decompression complained of discomfort in the infraspinatus area. His follow-up EMG after 6 months showed fibrillation potentials (1+) and positive sharp waves (1+) in the infraspinatus. The other patients’ EMG results showed no abnormality. The postoperative UCLA and VAS scores were improved in both groups, and there was no significant difference in the follow-up outcomes between the two groups.

**Conclusions:**

Patients with postersuperior MRCT and suprascapular neuropathy, decompression of suprascapular nerve at spinoglenoid notch didn’t lead to a better functional outcome with the repairment of rotator cuff. Arthroscopic rotator cuff repair could reverse the suprascapular neuropathy.

**Level of evidence:**

Level III.

## Introduction

Suprascapular neuropathy has become an increasingly recognized pathologic process and cause of shoulder pain and weakness over the past decades [1]. Andre Thomas has first proposed the suprascapular neuropathy in 1936 [2, 3]. The suprascapular nerve (SSN) is often compressed along its course, especially at the suprascapular notch and the spinoglenoid region [4, 5]. The mechanism of nerve injury can be roughly classified as compression or traction resulting from massive rotator cuff tears [6, 7], space-occupying leisongs [8–11], anatomic variations of suprascapular ligament [12] and spinoglenoid ligament [9, 13], distal clavicle [14] or scapular fractures [15], vascular compression [16, 17] and dynamic compression in athletes [17–20].

Rotator cuff tear can cause the suprascapular neuropathy, especially the massive rotator cuff tear (MRCT). The retracted rotator cuff may alter the course of the SSN, leading to a traction injury of the nerve [21]. The rotator cuff tears are classified into four categories according to Cofield: small tear < 1 cm, medium tear 1–3 cm, large tear 3–5 cm, and massive tear > 5 cm [22]. Lädermann et al. used a simple method (A-B-C-D) to classify the tear patterns: Type A: bone involvement; Type B:full thickness tendon lesion; Type C: musculotendinous junction leision; Type D: muscle insufficiency [23]. Each type has several subtypes. Lafosse et al. have reported the arthroscopic SSN release for repairing the rotator cuff [24, 25]. SSN entrapment of the suprascapular notch has been widely reported [3, 24, 26–29]. Until 1982, Aiello has described a patient with the SSN entrapment caused by the denervation of the infraspinatus at the spinoglenoid notch [30].

Albritton et al. have suggested the association between suprascapular neuropathy and MRCT [4]. They have reported that the retraction of supraspinatus and infraspinatus muscles changes the course of SSN, resulting in increased tension of SSN, and perhaps leading to severe fatty degeneration of the rotator cuff muscle. The role of SSN decompression is uncertain, and the indications for the patients with MRCT are undetermined due to the low (2%) prevalence [31]. Moreover, it still remains controversial whether the SSN can recover after the torn rotator cuff is repaired [3].

In the present study, we hypothesized that (1) patients receiving arthroscopic rotator cuff repair could have improved functional outcomes; (2) the symptoms of damaged SSN could be improved by repairing the rotator cuff; (3) Patients underwent the decompression of SSN at the spinoglenoid notch may have a better outcome in function and pain.

## Materials and methods

### Research population

**Fig. 1:**
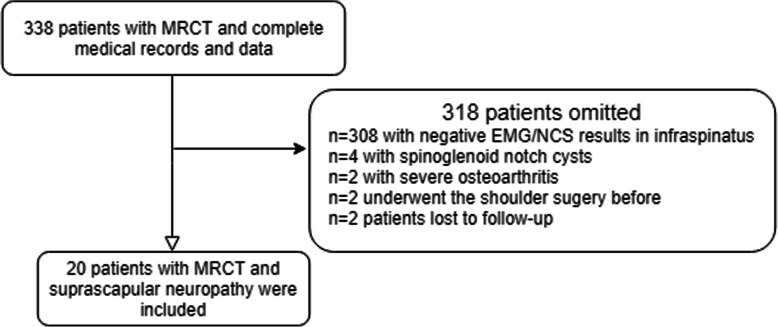
Flow chart of study population

From January 2013 to January 2019, 20/338 qualified patients with a minimum follow-up of 12 months who had posterosuperior MRCT and positive electromyography (EMG) /nerve conduction study (NCS) results were enrolled in the present analysis. All patients had symptoms of suprascapular neuropathy. The medical records of the above-mentioned patients were retrospectively analyzed. The rotator cuff tear was diagnosed by an experienced senior surgeon using the clinical examination and imaging examination. The suprascapular neuropathy was diagnosed by EMG/NCS. (Fig. 1).

The inclusion criteria were set as follows: (1) patients with MRCT in supraspinatus and infraspinatus muscles; (2) suprascapular neuropathy at “lower” position was diagnosed by EMG/NCS. The exclusion criteria were set as follows: (1) other shoulder pathology (rheumatoid arthritis or other inflammatory diseases of the shoulder); (2) patients who underwent the shoulder surgery before; and (3) patients who had the spinoglenoid notch cysts that compressed the SSN at the spinoglenoid notch. Patients were divided into two groups according to their surgical methods. Patients in the releasing group (A) underwent arthroscopic rotator cuff repair and decompression of SSN. Patients in the non-releasing group (B) were just treated by arthroscopic rotator cuff repair.

### Functional and supplementary assessments

Every patient received the UCLA and VAS questionnaire to assess the function and pain preoperatively and at 12 months after the operation. The total score of UCLA was 35, consisting of pain (10), function (10), Active forward flexion activity (5), strength (5), and patient satisfaction (5). It can be divided into three levels, excellent (34–35), good (29–33), and poor (< 29). The function score and active range of the shoulder were evaluated by our experienced doctor who didn’t attend the surgical operation. The postoperative range of motion (ROM) was examined in the following planes: forward elevation, external abduction, and external rotation (in abduction or at the side). UCLA and VAS scores were compared preoperatively and 12 months after the operation between the two groups. The average score in each group was calculated.

### Magnetic Resonance Imaging (MRI)

MRI was used to assess the structural integrity of the rotator cuff. The retraction degree of the rotator cuff was classified from the oblique frontal plane of MRI as previously described [32]. The fatty infiltration of the rotator cuff was classified from the parasagittal MRI image as previously described [33, 34]. All patients underwent an MRI examination preoperatively and 6 months after the surgery.

### Neurological examination

**Fig. 2:**
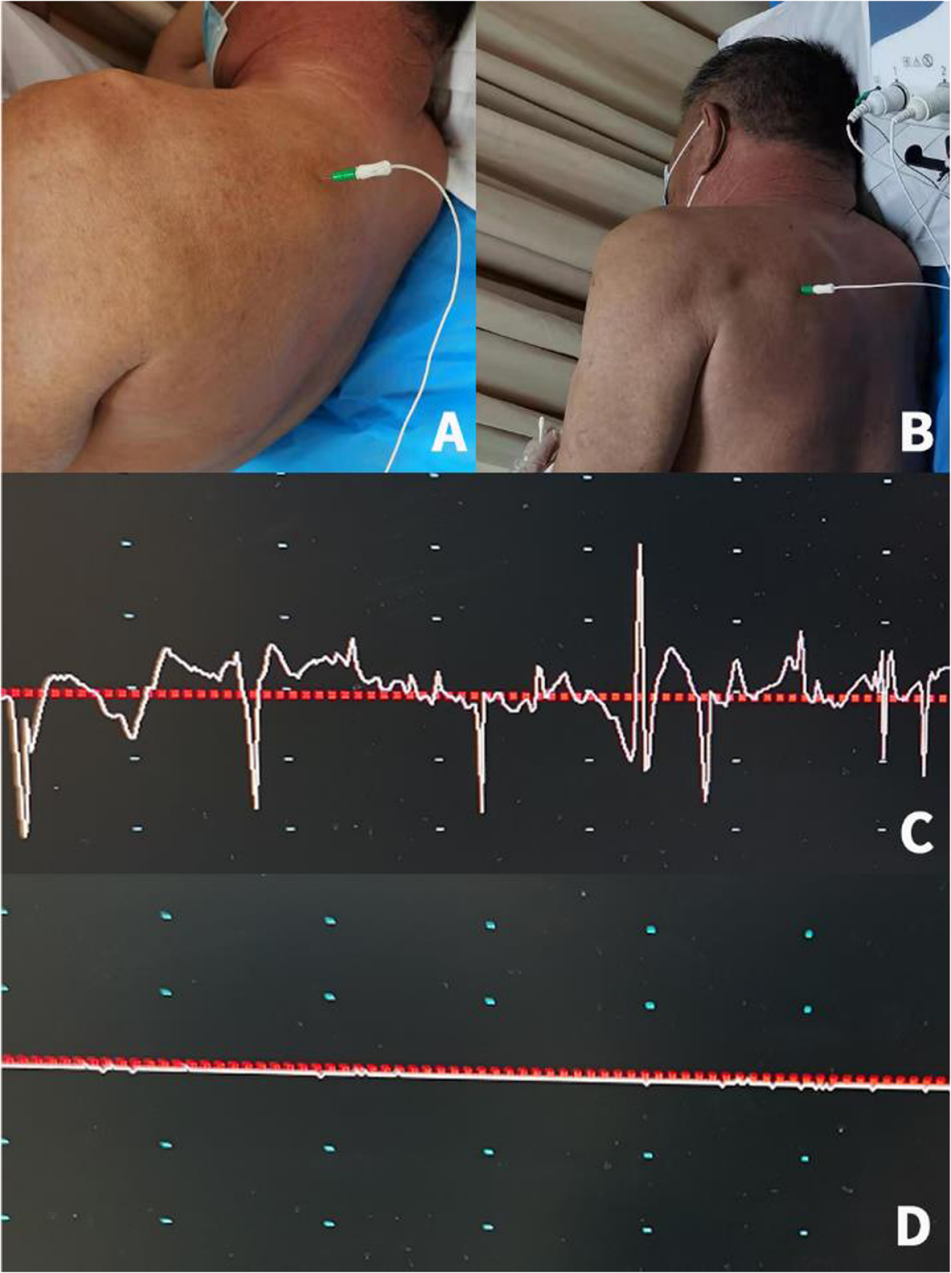
General information of EMG. **a** showed that the needle was inserted down to the bone in the supraspinous fossa. **b** showed that the needle was inserted down to the bone in the infraspinous fossa. **c** showed the preoperative EMG resutlts, we could see different degrees of fibrillation and positive sharp waves in the infraspinatus. **d** showed the EMG results after 6 months operation. There was no fibrilliation and positive sharp waves

The suprascapular neuropathy was evaluated by needle EMG and NCS. The same methods and diagnostic criteria were applied to all patients. Briefly, the patient was placed in the lateral position. The deltoid muscle, biceps, triceps, infraspinatus, and supraspinatus muscles were examined. The needle was inserted into the muscle to observe whether there were fibrillation potentials, positive sharp waves, complex repetitive discharge, and fasciculations in the spontaneous potential. Motor unit action potentials were qualitatively evaluated, and the recruitment pattern with the effort of muscle contraction was recorded. The vertical needle was inserted down to the bone in the supraspinous fossa and infraspinous fossa and then withdrawn slightly. The needle was inserted into the thickest part of the supraspinatus and infraspinatus (Fig. 2). To avoid interference from the trapezius, patients were asked to shrug their shoulders to stimulate the SSN. NCS included the latency and amplitude of compound muscle action potential during electrical stimulation. The surface electrode was placed at Erb’s point for electrical stimulation, and the same needle was adopted for data recording. Three measurements with different morphological potentials were performed to explore the maximum number of motor units in the nerve. The abnormal EMG indicated the presence of abnormal spontaneous activity, including the fibrillation potentials, positive sharp waves, complex repetitive discharge, and fasciculations. The criteria for abnormal NCS findings were as follows: supraspinatus muscle latency > 3.5 ms and an amplitude < 8 mV from peak to peak, or infraspinatus muscle latency > 4.5 ms and an amplitude < 8 mV from peak to peak [5, 31]. The above-mentioned abnormalities were used as the diagnostic criteria to identify the SSN neuropathy.

### Surgical procedures

Under general anesthesia with interscalene block, the patient was placed in the lateral decubitus position. The Spider shoulder positioner was used to regulate the traction force at 2.5 ~ 3.5 kg to keep the arm in a position of 20° flexion and 30° abduction. All surgical procedures were performed by an experienced senior shoulder surgeon in our hospital. Standard portals for all patients, such as anterior, posterior, lateral, and posterolateral, were made. All repairs were done using a transosseous equivalent technique with peek anchors. The suture methods depended on the types of rotator cuff tear. The long head of the biceps tendon was cut if it had an inflammatory reaction. The acromioplasty was carried out according to the acromion situation during operation. Finally, local anesthetics were injected into subacromial and intraarticular spaces.

**Fig. 3:**
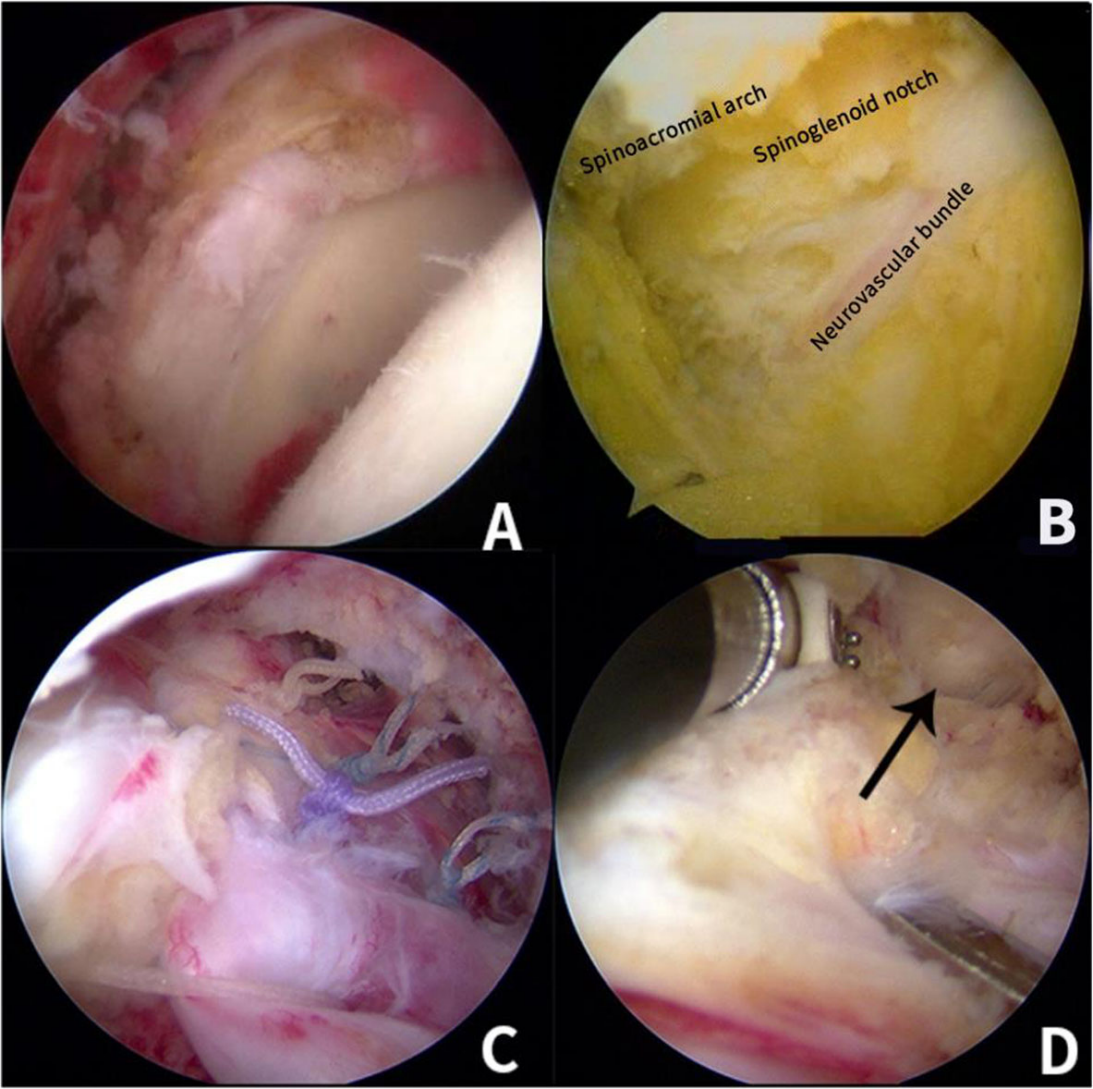
**a** shows the torn rotator cuff. **b** shows the neurovascular bundle. **c** shows that the rotator cuff structure was repaired by the anchors. In **d**, the black arrow shows the scapular spine, we used the radiofrequency probe to release both bursal and articular sides. The lifting pliers were used to grasp the edge of the tendon tear to facilitate arthroscopic releases

The patients in the releasing group underwent the decompression of SSN at the spinoglenoid notch before the repairment of the rotator cuff. A probe was used to locate the base of the scapular spine, and the neurovascular bundle was located at the spinoglenoid notch. The suprascapular neuropathy was believed to be caused by the traction of the nerve as the muscle retracted medially [4, 7, 21]. The spinoglenoid ligament was on the superior and lateral side of the nerve and didn’t directly compress the neurovascular bundle. The radiofrequency probe in the posterior portal was used to mobilize the tendon by performing both bursal and articular sided releases, resection of the coracohumeral ligament, and interval slide techniques. The lifting pliers were used to grasp the edge of the tendon tear, which could facilitate the arthroscopic releases [21].. We could see the spine of scapular and the neurovascular bundle. We could see the pulsating suprascapular artery that was a mark reminding us that the nerve was nearby. Using the probe to do blunt separation between the fascia of supraspinatus/infraspinatus and neurovascular bundle. Cutting the greater tension fascia around the neurovascular bundle. To avoid iatrogenic injury of the SSN, articular releases didn’t proceed more than 1.5 cm medial to the glenoid rim [21, 35]. After the decompression of SSN, the torn rotator cuff was repaired (Fig. 3).

### Postoperative rehabilitation

The operated shoulder was fixed at 40 degrees abduction for 6 weeks in both groups using the shoulder brace. The COX-2 drugs and nutritional neuro drugs were regularly used after the surgery. The pendulum and passive ROM exercises began 6 weeks after the surgery. After 8 weeks, the rehabilitation plan was modified according to the recovery situation of the patient, and the self-assisted passive and active ROM exercises were encouraged. Active strengthening exercises using an elastic band started 8 to 12 weeks postoperatively. Nearly full active ROM was allowed 4 months postoperatively.

### Statistical analysis

The Student’s t-test and Fisher’s test were used to compare the postoperative scores and demographic data of the two groups. A paired t-test was performed to compare the preoperative and postoperative scores in each group. All analyses were performed at the one-sided 5% significance. The SPSS software (version 22.0, IBM, Armonk, NY, USA) was used for the test.

## Results

All patients were diagnosed with MRCT and suprascapular neuropathy. Finally, 20 patients were recruited in our current study. The symptom of infraspinatus tear was severer compared with the supraspinatus tear from MRI and ECG/NCS, indicating that the compression of the SSN was probably at the spinoglenoid notch. Patients in the releasing group received the arthroscopic repair of the MRCT and decompression of SSN, and the other 10 patients in the non-releasing group were treated by arthroscopy only.

Table 1 shows a comparison of the baseline characteristics between the two groups. The patients were followed up for at least 12 months. All patients showed significant improvements in VAS scores at 12 months after operation compared with the preoperative scores (*P* < 0.05). The UCLA scores in group A increased from 11.56 ± 2.13 preoperative to 30.10 ± 1.91 postoperative (*P* < 0.05). The UCLA scores in group B increased from 11.30 ± 1.42 preoperative to 31.10 ± 2.02 postoperative (*P* < 0.05) (Table 2). There were no significant differences in UCLA scores (t = 1.136, *P* = 0.27) and VAS scores (t = 0.318, *P* = 0.75) between the two groups at 12 months after the operation.

**Table 1:** General characteristics of the study subjects

Demographics	Group A	Group B	P
Age (year)	59.90 ± 6.54	61.50 ± 6.15	0.56
Sex (female/male)	4/6	5/5	1.0
Symptom duration (month)	5.50 ± 3.14	5.00 ± 3.06	0.70
Side of involvement (left/right)	3/7	3/7	1.00
Involvement of dominant arm (number, %)	8, 80%	7, 70%	–
Preoperative shoulder ROM (°)			
Forward elevation	68.00 ± 8.08	66.60 ± 9.09	0.75
External abduction	46.60 ± 5.89	45.80 ± 6.46	0.73
External rotation	23.71 ± 9.61	24.9 ± 8.68	0.77
Retraction (Patte grade)			
Supraspinatus			1.00
(Grade 3/Grade 2)	5/5	6/4	
Infraspinatus			
(Grade 3/Grade 2)	7/3	7/3	1.00
Fatty infiltration (Goutallier grade)			
Supraspinatus			
(Grade 3/Grade 2/Grade1)	2/3/5	1/5/4	1.00
Infraspinatus			
(Grade 4/Grade 3/Grade2)	2/5/3	3/4/3	1.00

**Table 2:** Comparison of preoperative and postoperative (12 months after operation) UCLA and VAS scores between the two groups

	Group A			Group B		
	Preoperative	Postoperative	*P*	Preoperative	Postoperative	*P*
UCLA score	11.56 ± 2.13	30.10 ± 1.91	<0.05	11.30 ± 1.42	31.10 ± 2.02	<0.05
VAS score	7.40 ± 0.70	1.90 ± 0.74	<0.05	7.90 ± 0.87	2.00 ± 0.67	<0.05

**Fig. 4:**
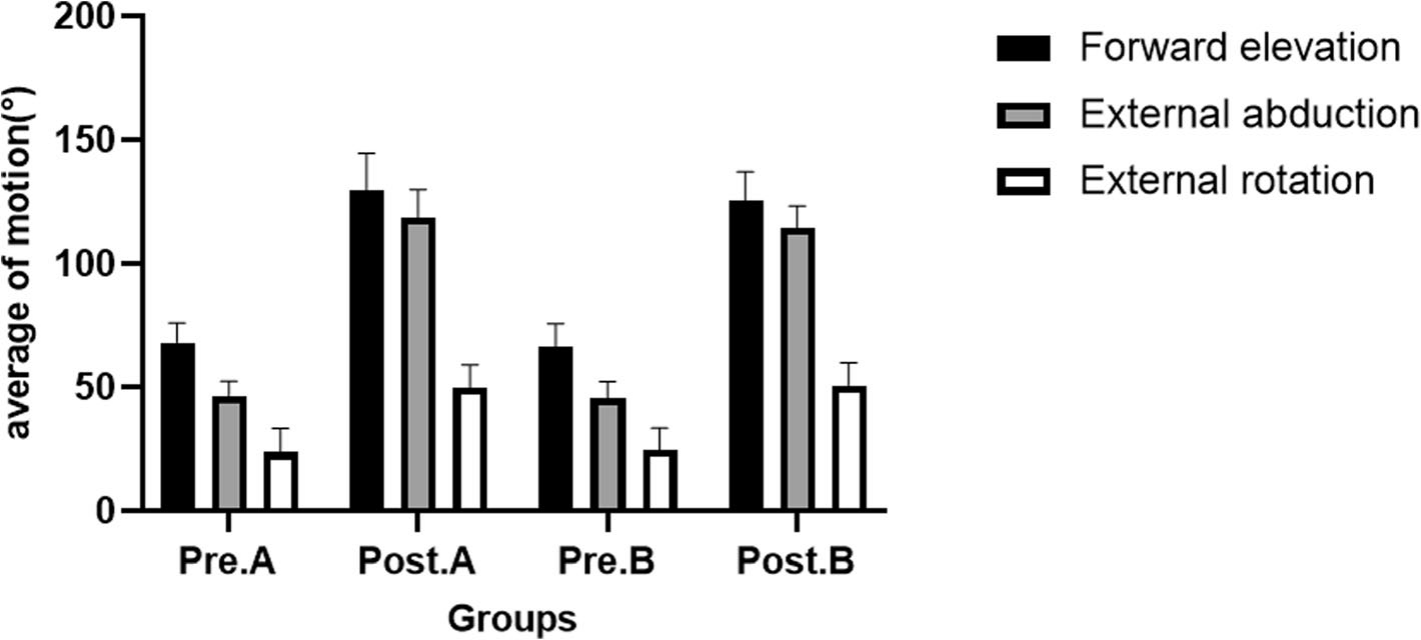
Comparisons of preoperative and postoperative ROM between the two groups

The ROM of forward elevation, external abduction, external rotation all significantly improved from preoperative levels (*P* < 0.05) (Fig. 4). All patients had great improvements in the strength, reaching the normal level at 12 months after operation. There were no significant differences in posteroperative mean forward elevation (129.50 ± 15.08 vs 125.50 ± 11.64, t = 0.664, *P* = 0.52), external abduction (118.60 ± 11.24 vs 114.30 ± 8.97, t = 0.946, *P* = 0.36), external rotation (49.70 ± 9.44 vs 50.80 ± 9.08, t = 0.266, *P* = 0.79) between the two groups.

The strength for abduction increased from a median of 3 (range, 2–3) preoperatively to a median of 5 (range, 4–5) postoperatively in group A (*P* < 0.05) and from a median of 3 (range, 2–4) preoperatively to a median of 5 (range, 3–5) in group B (*P* < 0.05). All 20 patients’ strength of external rotation returned to normal. The external rotation resistence strength test and external rotation lag sign were negative in all 20 patients.

However, one patient in the releasing group complained of discomfort in the infraspinatus area after the operation. He felt a little bit stiff at the beginning of the external rotation of the shoulder joint. The symptom disappeared at 6 months after the operation.

All the patients felt pain in the posterolateral aspect of the shoulder, and the pain could radiate to the neck and deltoid. A weakness of shoulder abduction and muscle atrophy were often complained by the patients.

**Fig. 5:**
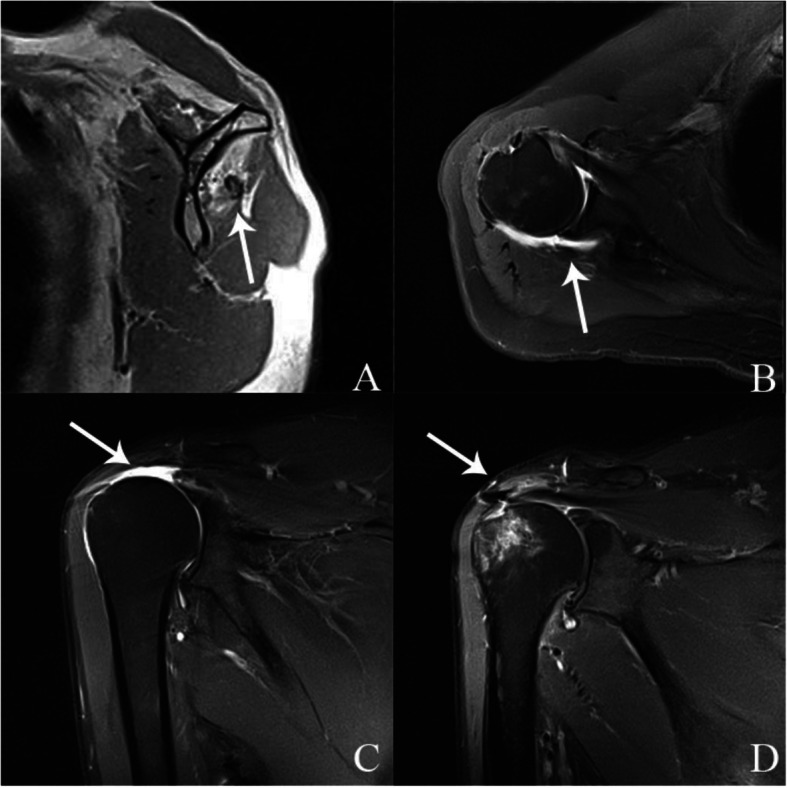
**a** shows a patient in our study with more fatty infiltration in infraspinatus (white arrow) compared with supraspinatus. **b** shows patients with severe infraspinatus retraction (white arrow). **c** shows MRCT in supraspinatus (white arrow). (Patte grade 3). **d** shows that the supraspinatus was healed well at 6 months after the operation

At 6 months after the operation, all patients underwent an MRI examination. One patient in the non-releasing group showed the retear of the rotator cuff. However, he didn’t complain of pain and terrible activities. The remaining 19 patients had no retear. The MRI showed that fatty infiltration didn’t develop further, while it was not reversed either. (Fig. 5) At the last follow-up (range, 12-24 months), we founded that there were 2 patients had rotator cuff retear in group A and 1 patients retear in group B. One patient underwent revere total shoulder arthroplasty surgery due to severe rotator cuff retration and fatty infiltration. Others adopted conservative observation because they had no pain and they were satisfied with the function. The retear rate of group A and group B were 30% (3/10) and 20% (2/10) respectively.

Preoperative EMG showed that there were different degrees of fibrillation and positive sharp waves in the infraspinatus (Table 3). The duration of the motor unit potential in the infraspinatus was prolonged, and the amplitude was more than 5 mV. It also showed that the polyphase wave was increased, and the recruitment pattern was weakened. In NCS, the latency of compound muscle action potential in the infraspinatus was prolonged, and its amplitude was decreased. These results suggested that the infraspinatus branch of the SSN was injured. Five of twenty patients also had abnormal results in EMG and NCS of the supraspinatus muscle, while the symptoms were less severe compared with the infraspinatus muscle. At 6 months after the operation, the EMG results showed no abnormality in all patients. The latency and amplitude of cMAP in the infraspinatus muscle returned to normal range.

**Table 3:** Preoperative EMG/NCS results of the two groups

Rotator cuff	Spontaneous potential		Motor unit potential		recruitment pattern	Compound muscle action potential	
	Fibrillation potentials (0, 1 + , 2 + , 3+)	Positive sharp waves (0, 1 + , 2 + , 3+)	Increased duration (n)	Increased amplitude (n)	Weaken (n)	Latency (^−^x ± s, ms)	Amplitude (^−^x ± s, mV)
Releasing group (A)							
supraspinatus	5, 5, 0, 0	6, 4, 0, 0	2	2	2	3.07 ± 0.82	10.08 ± 1.98
infraspinatus	0, 2, 5, 3	0, 2, 7, 1	10	9	10	7.75 ± 1.09	7.74 ± 1.22
Non-releasing group (B)							
supraspinatus	5, 4, 1, 0	5, 5, 0, 0	3	2	2	3.30 ± 0.87	9.90 ± 1.78
infraspinatus	0, 1, 6, 3	0, 2, 5, 3	10	9	9	7.63 ± 1.15	7.41 ± 1.28

## Discussion

In the present study, we aimed to assess whether releasing the SSN at the spinoglenoid notch could affect the prognosis of posterosuperior MRCT. At first, we hypothesized that patients underwent the decompression of SSN at the spinoglenoid notch may have a better outcome in function and pain. But from the results, we found that after the MRCT was repaired, patients could get an ideal recovery. The strength for abduction and external rotation improved significantly from preoperative to postoperative. The ROM of forward elevation, external abduction, external rotation all significantly improved from preoperative levels. However, the decompression of SSN didn’t show a better effect on the functional outcome. One patient in the releasing group felt stiffness at the beginning of the external rotation. We considered that the decompression of soft tissue and the excision of synovial tissue might result in scarring [36] or iatrogenic injury [37]. The new scar could affect muscle activity and even cause nerve compression. The retear rate of group A and group B were 30 and 20% respectively. The retear rate was lower than that reported by other scholars [38, 39]. We thought the reason was that we changed the lifestyle of the patients. We forbided them to do the movements that could cause rotator cuff tear.

The reason for the suprascapular neuropathy is the compression of the nerve along its course [4, 5]. Al-Redouan et.al reported a detailed topographical study of the suprascapular canal [40]. They concluded that the suprascapular nerve passes through the suprascapular canal within 5 intervals (pre-entrance, entrance, passage, exit, post-exit) and each interval carry its own extent of variations and potential causes of entrapment. Within the passage interval, the most common cause was rotator cuff tear. The motor branch of suprascapular nerve could be pulled medially by the displaced supraspinatus and its fascia [1, 41]. Repetitive arm adduction exercises makes the athletes very susceptible to injury, especially in weight lifters [17–19]. The nerve was compressed against the spinoglenoid fossa dynamically [1]. With the exit interval, the ganglion cyst is the most frequently reported etiology of suprascapular nerve entrapment [8–10]. The anatomical variations of spinoglenoid ligament like widely fanned could compress the suprascapular nerve [9, 13]. For the post-exit interval, the rotator cuff tear affecting the infraspinatus muscle could lead to suprascapular nerve entrapment caused by the nerve traction injury [8, 42, 43]. Knowledge of these structural variations can help us determine the cause of the neuropathy. We could determinate the location of injury according to the symptom. Weakness and atrophy of both the supraspinatus and infraspinatus indicate the entrapment at the entrance or passage interval. Weakness and atrophy of the infraspinatus suggest the injury at the exit intervals [28].

The suprascapular neuropathy was considered to be an exclusive diagnosis. With the improvement of diagnostic techniques, surgeons began to pay attention to suprascapular neuropathy. Collin et al. have performed a prospective EMG study and shown that there is a low prevalence of SSN neuropathy, and only 2% of the patients are diagnosed by the EMG [31]. It confirmed the results of Vad et.al with an incidence of 8% (2/25) [44]. In our study, the incidence of suprascapular neuropathy in the setting of posterosuperior MRCT was 8.7% (30/338). The incidence of suprascapular neuropathy in the setting of MRCT and moderate to severe fatty infiltration was 38% (7/26) in study of Costouros [21]. The incidence was inconsistent due to the different inclusion criteria of studies. In conclusion, we thought the incidence of suprascapular neuropathy in the setting of MRCT was below 10%. However, with the aggravation of fatty infiltration and retraction of rotator cuff, the incidence of neuropathy will increase.

For patients with suprascapular neuropathy in the setting of MRCT, there is no unified treatment standard at present [6, 20, 27, 28]. For the treatment of suprascapular neuropathy, Lafosse et al. described the technique of arthroscopic transverse scapular ligament dissection in 2007 and nine out of ten patients had satisfactory outcomes [24]. Most reports have focused on the suprascapular neuropathy in the setting of MRCT [1, 27, 31, 45]. It has been demonstrated that repairment of rotator cuff could reverse the suprascapular neuropathy [21]. Yamakado et al. have evaluated 67 patients with posterosuperior MRCT who undergo complete repair of the torn rotator cuff [27]. A total of 36 patients receive the SSN decompression by removing the transverse scapular ligament. However, the functional outcomes of the SSN decompression group are not significantly different from the group just retreated with the arthroscopic rotator cuff repair. They have concluded that decompression of SSN by cutting the transverse scapular ligament had no significant effect on the prognosis of posterosuperior MRCT. In the study of Nolte et.al, they concluded that in the absence of major concomitant glenohumeral pathology, arthroscopic SSN decompression at the suprascapular notch and/or spinoglenoid notch could lead to a significant improvement in functional outcomes [28]. Sachinis et al. reported that they were conducting a randomized controlled trial about 42 patients with MRCT and suprascapular neuropathy [46]. One objective of their trial was to determine the effect of SSN decompression. We have known that rotator cuff repair with SSN decompression at suprascapular notch doesn’t lead to significant improvement compared to rotator cuff repair alone form the study of Yamakado et al. [27].

A study has found that transosseous double-row repair of the tendons can effectively restore the anatomic nerve course. Costouros et al. have demonstrated that either partial or complete arthroscopic repair of the posterior MRCT can lead to satisfactory outcomes in the EMG study and function 6 months later [21]. They thought that repaired the infraspinatus in a more superior and lateral position could restore the compressed nerve. We believed that repairing the torn rotator cuff could relieve the tension on the nerve around the scapular, which was consistent with Burkhart’s concept of partial arthroscopic repair of MRCT [47].

In our study, we aimed to determine the effect of decompression of SSN at spinoglenoid notch and fossa. We selected the patients with suprascapulalr neuropathy at “lower” position according to the symptom, preoperative MRI and EMG/NCS [28]. So we performed the decompression at the spinoglenoid notch and fossa not the suprascapular notch. Through our follow-up data results, we concluded that decompression at spinoglenoid notch and fossa didn’t lead to a better outcome if the rotator cuff was repaired. The result was the same as Yamakado confirmed at the suprascapular notch [27]. This study further proves that for the suprascapular neuropathy caused by MRCT, repairment of rotator cuff alone could reverse the neuropathy and lead to a good functional outcome.

There are several limitations in our study. As a retrospective study, the small sample size is an additional weakness that is hard to guarantee more accurate and credible results. The diagnosis standard of suprascapular neuropathy needs further validation. It lacked unified diagnostic criteria. And we wondered if the effect of decompression of SSN might be masked by repairment of rotator cuff. So the effect of decompression needs to be further confirmed. Despite these limitations, the relationship between the MRCT and suprascapular neuropathy still needs to be further clarified in future studies.

## Conclusions

For the patients with posterosuperior MRCT and suprascapular neuropathy, we concluded that decompression of suprascapular nerve didn’t lead to a better functional outcome with the repairment of rotator cuff. Arthroscopic rotator cuff repair could reverse the suprascapular neuropathy.

## Data Availability

All data generated or analysed during this study are included in this article.
